# Trauma outcomes differences in females: a prospective analysis of 76 000 trauma patients in the Asia-Pacific region and the contributing factors

**DOI:** 10.1186/s13049-025-01342-1

**Published:** 2025-02-24

**Authors:** Mohamad Iqhbal Bin Kunji Mohamad, Sabariah Faizah Jamaluddin, Norhaiza Ahmad, Arifah Bahar, Zarina Mohd Khalid, Nuraina Aqilah Binti Mohd Zaki, Nurul Azlean Norzan, Sang Do Shin, Goh E. Shaun, Wen-Chu Chiang, Kentaro Kajino, Kyoung Jun Song, Do Ngoc Son

**Affiliations:** 1 Faculty of Medicine, Universiti Teknologi MARA, UITM Sungai Buloh Campus, Jalan Hospital, Sungai Buloh, Selangor 47000 Malaysia; 2 Department of Mathematical Sciences, Faculty of Science, Universiti Teknologi Malaysia, Johor Bahru, Malaysia; 3 UTM-Centre for Industrial and Applied Mathematics, Universiti Teknologi Malaysia, Johor Bahru, 81310 Malaysia; 4 Emergency and Trauma Department, Sungai Buloh Hospital, Jalan Hospital, Sungai Buloh, Selangor 47000 Malaysia; 5 Department of Emergency Medicine, Seoul National University College of Medicine and Hospital, Seoul, Korea; 6 Emergency Department, Woodlands Health Campus, Houston, Singapore; 7 Department of Emergency Medicine, National Taiwan University Hospital, Taipei, Taiwan; 8 Department of Emergency and Critical Care Medicine, Kansai Medical University, Hirakata, Osaka Japan; 9 Department of Emergency Medicine, Seoul National University Boramae Medical Center, Seoul, South Korea; 10 Center for Emergency Medicine, Bach Mai Hospital, Hanoi, Vietnam

**Keywords:** Trauma mortality, Asian females, Glasgow outcome scale, Modified Rankin’s scale, Trauma in-hospital mortality, Trauma sex differences, Asia-Pacific trauma, Trauma biological changes, Trauma physiological changes

## Abstract

**Background:**

Trauma is a leading cause of mortality, particularly in low and middle-income countries. While extensively studied in North America and Europe, data from the Asia-Pacific are limited. An important area of research is the difference in trauma outcomes, which are theoretically noted to be better among females. However, the clinical findings are inconclusive among Asians. This study examines sex-based differences in trauma outcomes in Asia Pacific, focusing on in-hospital mortality and functional recovery at discharge.

**Methods:**

This observational study, from the Pan-Asia Trauma Outcomes Study (PATOS), included 76,645 trauma patients from 12 Asian Pacific countries. We analysed in-hospital mortality and functionality at discharge using the Glasgow Outcome Scale (GOS) and the modified Rankin Scale (mRS). Logistic regression models were built to test the association of sex on the outcomes.

**Results:**

Males exhibited higher in-hospital mortality (1.6%) compared to females (1.06%) ( *p* < 0.001). Adjusted logistic regression models showed that the female sex is not independently associated with in-hospital mortality. Females have a better functional outcome at discharge for patients younger than 50 years with ISS < 16. However, no significant differences existed between those > 50 years and ISS > 15.

**Conclusion:**

This study indicates no difference in the general trauma outcomes in the Asia Pacific between females and males. Although younger females with less severe injuries had better functional outcomes, this advantage disappeared in severe injuries and those over 50 years. These results align with some previous studies, and understanding the nuances may lead to more tailored trauma care, potentially improving patient outcomes.

## Introduction

Globally, trauma is a leading cause of mortality, contributing to over 4.4 million deaths annually, with a disproportionate burden in low- and middle-income countries [1]. While Northern America and European countries have contributed extensively to the body of trauma literature, there remains a relative lack of data from Asian countries despite their significant trauma burden [2, 3]. Among the factors that contribute to trauma mortality, aside from the mechanism and the severity of the injury, are the healthcare system, comorbidities, age, and likely sex differences [4–7]. Understanding the nuances of sex-based differences in trauma outcomes could advance personalised trauma management, inspire research to improve trauma care, and facilitate better decision-making and prognostication.

The physiological response to trauma, characterised by initial acute inflammatory response and subsequent immunosuppression, may have sex-specific differences [8]. Laboratory research has demonstrated differences in outcomes after major trauma, sepsis, and haemorrhage in females [9, 10]. Hormonal variations, particularly estrogen and testosterone, could influence the immune and overall physiological responses to traumatic injuries in animal studies [11, 12]. These findings are supported by studies indicating that genetic factors, such as X-chromosome-linked polymorphisms in the innate immune response to sepsis, are associated with poor outcomes, which can be a plausible mechanism for sex-based differences in outcomes following injuries [9, 13, 14].

Clinical studies, however, have presented inconsistent results. An analysis of 36,000 patients with blunt trauma in Germany showed no difference in outcome between sexes, although the male sex was an independent negative predictor of morbidity [15]. In the Netherlands, a study involving 7,000 patients at level 1 trauma centres showed that sex was not an independent predictor for in-hospital mortality. Still, males were more likely to be admitted into the ICU. Two Chinese studies showed that females had a lower risk of mortality than males after severe blunt trauma [3, 16]. Notably, studies specific to Asian populations have suggested potential regional differences in these patterns, a hypothesis that has yet to be fully explored with large-scale data [17].

Our study aimed to bridge this gap by analysing the differences in trauma outcomes between sexes in a diverse patient population from the Asia-Pacific region. We focused on in-hospital mortality rates and functionality at discharge, using the Glasgow Outcome Scale (GOS) and modified Rankin’s Scale (mRS) to better understand the nuances of recovery. Our ultimate goal is to enhance the understanding of trauma outcomes and to contribute to the development of tailored approaches to trauma care that consider sex as a significant factor.

## Methods

### Study setting

The data for this study were extracted from The Pan-Asia Trauma Outcomes Study (PATOS), an international multicenter observational research network founded in 2013. The primary aim of PATOS is to create a collaborative, standardised registry of injury patients across Asia, focusing on the processes and outcomes of trauma cases transported by emergency medical service (EMS) providers. The hospital’s emergency departments coordinate data collection in various Asian countries that receive trauma patients from EMS. The collected data encompass five key categories:1) injury epidemiologic factors, 2) EMS factors, 3) emergency department care factors, 4) hospital care factors, and 5) trauma system factors.

The study recruited hospitals from twelve countries, with the electronic data capture (EDC)system hosted by the Study Coordinating Centre at Seoul National University Hospital, Korea. Detailed descriptions of PATOS and its methodologies are available in other articles [18]. We retrospectively analysed the data collected between November 2015 and March 2021.

### Inclusion criteria and patient population

We included trauma patients aged 16 years and above in the study. Patients were excluded if their data were incomplete or if their injuries were attributable to drowning or poisoning to maintain the focus on trauma from physical causes.

### Objective and outcomes assessment

The outcomes assessed were the mortality and functional status. Functionality was assessed using validated scales, namely the Glasgow Outcome Score (GOS) and the modified Rankin’s Scale (mRS). Although both scales were initially developed for assessing traumatic brain injury and cerebrovascular accidents, they have been advocated for use in general trauma. Various studies have utilised these scales to assess outcomes beyond brain injuries [19–23]. Poor functionality is defined as a GOS score of 2–3 or an mRS score of 4–5 based on established correlations between the two scoring systems in the literature, reflecting significant disability [24].

### Variable consideration and rationale

Independent variables include age (categorised as < 50 and ≥ 50). This division was based on the hypothesis that trauma response differences between sexes could be due to hormonal changes, which are related to menopausal status. The average menopausal age among females in the Asia Pacific region is 50 years old [25, 26]. Other variables are the mechanism of injury, which was divided into penetrating and non-penetrating; anatomical location of injuries (divided into head, neck, face, thorax, abdomen, spine, upper extremities, lower extremities and others); vital signs upon admission, which included systolic blood pressure(SBP) and respiratory rate (RR), admission Glasgow Coma Scale (GCS), and admission Injury Severity Score (ISS).

### Statistical analysis

The descriptive data were presented as categorical data, written as frequencies and percentages. Differences between female and male groups were analysed using the Pearson chi-square test or Fisher’s exact test for the categorical data. A *p*-value of < 0.001 was taken as the level of significance. Univariate analysis was performed to identify gross differences in the outcomes between sexes. Subsequently, cases with in-hospital mortality, poor GOS, and poor mRS upon discharge were extracted for further analysis.

Multivariable logistic regression models were constructed to investigate the association between independent variables and outcomes. Variable selection for inclusion in the models was based on clinical relevance and statistical significance, with a conventional threshold of *p* < 0.05 employed. We applied the Enter and Forward stepwise elimination methods to refine the models, carefully considering issues such as multicollinearity. In each model, the “female” was tested to assess the association of females as the independent variable and the outcomes. Another regression model was built for patients under 50 years using similar methods to investigate age-specific effects. The final model presented as an adjusted odds ratio ( aOR ) with a *p*-value < 0.001 as the significance level and a 99% confidence interval. All the analyses were done with Python software ( version 3.8 ).

## Results

### Demography and injury characteristics

**Fig. 1:**
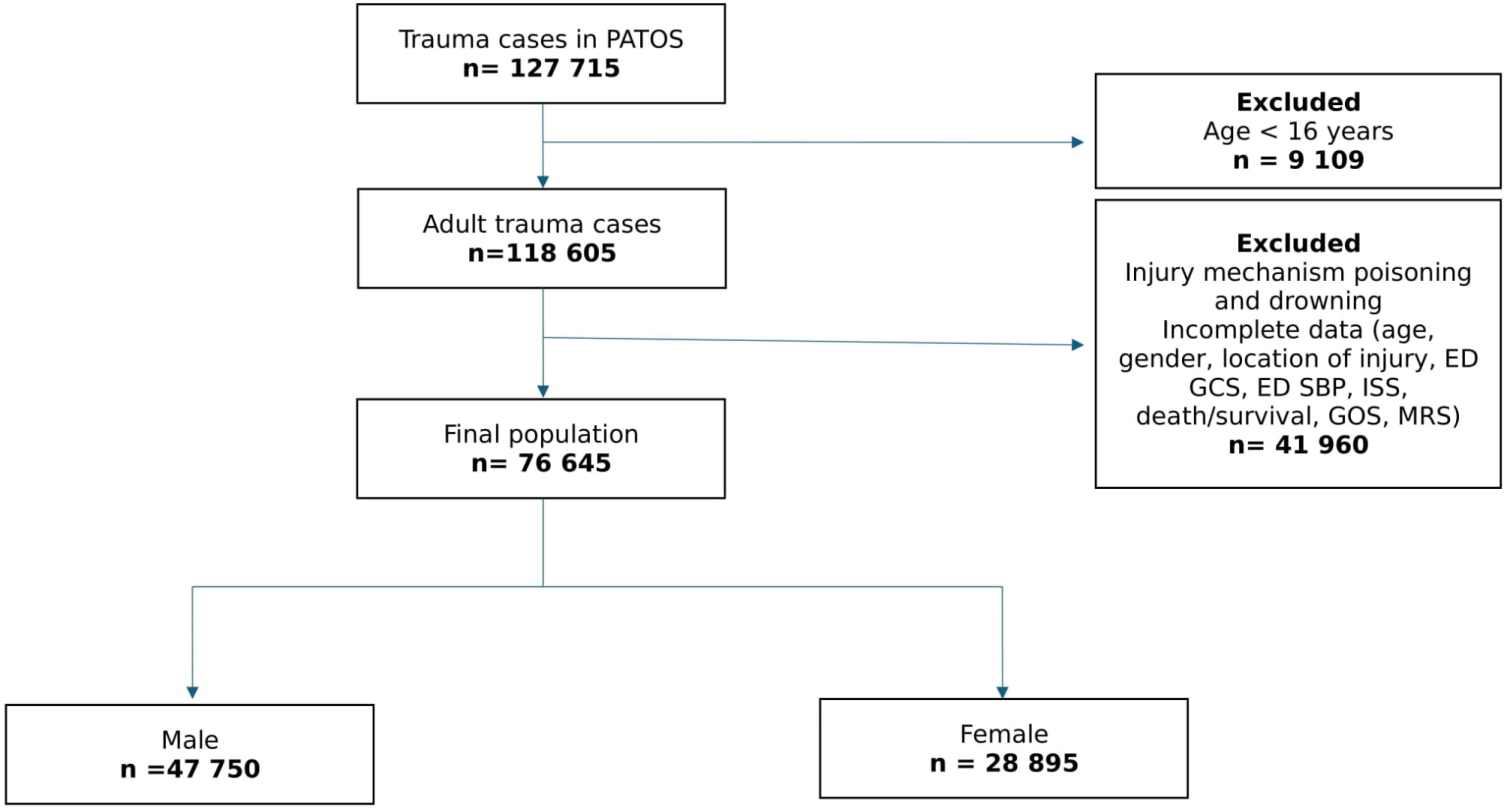
The flow of study

The final cohort comprised 76,645 trauma patients, with males constituting 62.3% (*n* = 47,750) and females 37.7% (*n* = 28,895) (Fig. 1). As demonstrated in Table 1, the age distribution highlighted a significant sex disparity, with females more frequently represented in the ≥ 50 age group (60.13%) compared to males (44.87%) (*p* < 0.001). Trauma type also varied between sexes; 95.51% of females experienced blunt trauma compared to 93.65% of males (*p* < 0.001). Anatomically, males predominantly sustained injuries to the head, face, thorax, abdomen, and upper extremities, whereas females more frequently suffered injuries to the lower extremities and spine (*p* < 0.001). Admission systolic blood pressure ( SBP ) < 90 mmHg, Glasgow Coma Scale ( GCS ) and Injury Severity Score ( ISS) were significantly associated with sex ( *p* < 0.001).

**Table 1:** Characteristics of trauma patients based on sex in all age group (*n* = 76645)

Variable	Categories		Females *n* = 28,895	%	Males *n* = 47,750	%	Total *n* = 76,645	*P* value
**Age**	< 50		11,521	39.87	26,324	55.13	37,845	**< 0.001**
	≥ 50		17,374	60.13	21,426	44.87	38,800	
**Mechanism of Injury**	Blunt ( Total)		27,598	95.51	44,718	93.65	72,316	**< 0.001**
		Traffic injury	10,793	37.35	21,740	45.53	32,533	
		Fall/slip down	12,754	44.14	14,816	31.02	27,570	
		Assault	2655	9.19	6214	13.01	8869	
		Others	1396	4.83	1948	4.08	3344	
	Penetrating Injury		1297	4.49	3032	6.35	4329	
**Anatomical Location of Injury**	Head		8453	29.25	15,879	33.25	24,332	**< 0.001**
	Face		5646	19.54	13,136	27.51	18,782	**< 0.001**
	Neck		1732	5.99	3064	6.42	4796	**0.020**
	Thorax		3243	11.22	7431	15.56	10,674	**< 0.001**
	Abdomen		1918	6.64	4074	8.53	5992	**< 0.001**
	Spine		2449	8.48	3265	6.84	5714	**< 0.001**
	Upper Extremity		6480	22.43	12,433	26.04	18,913	**< 0.001**
	Lower Extremity		11,517	39.86	15,547	32.56	27,064	**< 0.001**
	Other		162	0.56	361	0.76	523	**0.002**
**Admission Physiological Parameters**	SBP < 90		457	1.58	1150	2.41	1607	**< 0.001**
	RR > 20		1723	5.96	4258	8.92	5981	**0.795**
**Admission Glasgow Coma Scale (GCS)**	13–15		28,088	97.21	45,384	95.05	73,472	**< 0.001**
	9–12		403	1.39	1082	2.27	1485	
	3–8		404	1.40	1284	2.69	1688	
**Admission Injury Severity Score (ISS)**	ISS < 9		21,660	74.96	34,957	73.21	56,617	**< 0.001**
	ISS 9–15		5230	18.10	7415	15.53	12,645	
	ISS 16–25		1474	5.10	3881	8.13	5355	
	ISS > 25		531	1.84	1497	3.14	2994	

SBP, Systolic Blood Pressure; RR, Respiratory Rate

### The overall in-hospital mortality and poor GOS and mRS at discharge

As demonstrated in Table 2, the overall in-hospital mortality rate was 1.4%, with males exhibiting a higher mortality rate (1.6%) compared to females (1.06%, *p* < 0.001). The females contribute 28% ( *n* = 307) of the total mortality rate. Although females had a slightly higher rate of poor functional outcomes in the Glasgow Outcome Scale (GOS) and Modified Rankin’s Scale (mRS) at discharge, these differences were not statistically significant. The subgroup analysis of those with ISS > 15 revealed an overall mortality rate of 9.55%, with 8.78% in females and 9.81% in males ( *p* = 0.176).

**Table 2:** The mortality and functional outcome of trauma between sexes in all age groups

	Total	Females (%)	Males (%)	*P* -value
**The overall in-hospital mortality**	1072(1.4%)	307 (1.06%)	765 (1.6%)	< 0.001
**The overall poor discharge GOS** ^**b**^	5170 (6.75%)	1990 (6.89%)	3180 (6.66%)	0.224
**The overall poor discharge mRS** ^**b**^	6677 (8.81%)	2576 (8.99%)	4101 (8.71%)	0.182

GOS, Glasgow Outcome Scale; MRS, Modified Rankin’s Scale

^a^ Defined as GOS score of 2–3

^b^ Defined as mRS score of 4–5

### Characteristics of in-hospital mortality and poor GOS and mRS upon discharge based on sex

Sex differences were significant in in-hospital mortality, stratified by age groups, blunt mechanism of injury, and anatomical location of injury at the head (*p* < 0.001) (Table 3). Similarly, analysis of poor GOS and mRS at discharge revealed significant sex differences, particularly in age < 50, penetrating mechanism of injury, anatomical location, respiratory rate on admission, GCS 13–15, and ISS 9–15 (*p* < 0.001).

**Table 3:** Characteristics of in-hospital mortality and functionality outcomes in trauma based on sex in all age groups

Variable	Categories	Mortality		*P* value	Poor Discharge GOS^a^		*P* value	Poor Discharge mRS^b^		*P* value
		Females *n* = 307 (%)	Males *n* = 765 (%)		Females *n* = 1990 (%)	Males *n* = 3180 (%)		Females *n* = 2576 (%)	Males *n* = 4101 (%)	
**Age**	< 50	49 ( 15.96)	212 (27.71)	**< 0.001**	265 (13.31)	1089 (34.25)	**< 0.001**	429 (16.65)	1633 (39.82)	**< 0.001**
	≥ 50	258 (84.04)	553 (72.29)	**< 0.001**	1725 (86.68)	2091 (65.75)	**0.871**	2147 (83.35)	2468 (60.18)	**0.186**
**Mechanism of Injury**	Blunt	304 (99.02	754 (98.56)	**< 0.001**	1974 (99.19)	3119 (98.08)	**0.406**	2557 (99.26)	3987 (97.22)	**0.180**
	Penetrating Injury	3 (0.98)	11 (1.44)	**0.685**	16 (0.80)	61 (1.92)	**< 0.001**	19 (0.74)	114 (2.78)	**< 0.001**
**Anatomical Location Of Injury**	Head	193 (62.87)	559 (73.07)	**< 0.001**	548 (27.54)	1418 (44.59)	**< 0.001**	608 (23.60)	1554 (37.89)	**< 0.001**
	Face	61 (19.87)	174 (27.75)	**0.591**	240 (12.06)	628 (19.75)	**0.112**	254 (9.86)	757 (18.46)	**< 0.001**
	Neck	23 (7.49)	55 (7.19)	**0.966**	61 (3.07)	220 (6.92)	**< 0.001**	71 (2.76)	224 (5.46)	**< 0.001**
	Thorax	85 (27.69)	258 (33.73)	**0.562**	305 (15.33)	825 (25.94)	**0.007**	333 (12.93)	959 (23.38)	**< 0.001**
	Abdomen	71 (23.13)	177 (23.14)	**0.526**	185 (9.29)	489 (15.38)	**0.007**	244 (9.47)	612 (14.92)	**0.014**
	Spine	31 (10.10)	71 (9.28)	**0.481**	190 (9.55)	467 (14.69)	**< 0.001**	213 (8.27)	480 (11.70)	**< 0.001**
	Upper Extremity	40 (13.03)	102 (13.33)	**0.420**	274 (13.77)	566 (17.79)	**0.309**	290 (11.26)	687 (16.75)	**0.002**
	Lower Extremity	120 (39.09)	225 (29.41)	**0.337**	1324 (66.53)	1531 (48.14)	**< 0.001**	1799 (69.84)	2144 (52.28)	**< 0.001**
	Other	8 (2.61)	23 (3.01)	**0.055**	12 (0.60)	31 (0.97)		14 (0.54)	39 (0.95)	**0.510**
**Admission Physiological Parameters**	SBP < 90	81 (26.38)	186 (24.31)	**0.497**	103 (5.16)	279 (8.77)	**0.578**	112 (4.35)	279 (6.80)	**0.864**
	RR > 20	86 (28.01)	284 (37.12)	**0.017**	237 (11.90)	718 (22.58)	**0.001**	241 (9.36)	755 (18.41)	**< 0 0.001**
**Admission Glasgow Coma Scale (GCS)**	13–15	131 (42.67)	262 (34.25)	**0.576**	1747 (87.79)	2480 (77.99)	**< 0.001**	2335 (90.64)	3417 (83.32)	**< 0.001**
	9–12	33 (10.75)	70 (9.15)	**0.296**	104 (5.23)	247 (7.77)	**0.161**	110 (4.27)	243 (5.93)	**0.029**
	3–8	143 (46.58)	433 (56.60)	**0.051**	139 (6.98)	453 (14.25)	**0.922**	131 (5.09)	441 (10.75)	**0.512**
**Admission Injury Severity Score (ISS)**	ISS < 9	51 (16.61)	81 (10.59)	**0.999**	457 (22.96)	747 (23.49)	**0.851**	766 (29.74)	1313 (32.02)	**0.181**
	ISS 9–15	80 (26.05)	156 (20.39)	**0.022**	1088 (54.67)	1158 (36.42)	**< 0.001**	1380 (53.57)	1545 (37.67)	**< 0.001**
	ISS 16–25	93 (30.29)	262 (34.25)	**0.604**	300 (15.08)	830 (26.10)	**0.379**	226 (8.77)	670 (16.34)	**0.063**
	ISS > 25	83 (27.04)	266 (34.77)	**0.292**	145 (7.29)	445 (13.99)	**0.159**	204 (7.92)	573 (19.97)	**0.744**

GOS, Glasgow Outcome Scale; MRS, Modified Rankin’s Scale; SBP, Systolic Blood Pressure; RR, Respiratory Rate

^a^ Defined as GOS score of 2–3

^b^ Defined as mRS score of 4–5

### The logistic regression model in assessing the associations with outcomes

The logistic regression models showed that several variables were associated with poor outcomes. In the overall cohort, the female sex was not independently associated with in-hospital mortality or poor functional status at discharge in the general group analysis, with the adjusted odds ratios near one and *p*-values > 0.001 (Table 4). However, in the subgroup analysis of patients under 50 years, the female sex is associated with lower odds of poor GOS( aOR 0.63, 99% CI 0.51 to 0.78, *p* < 0.001) and poor mRS upon discharge ( aOR 0.72, 99% CI 0.62 to 0.83 *p* < 0.001), although there was no difference the in-hospital mortality (*p* = 0.907) (Table 5). As presented in Table 6, in the subgroup of patients under 50 years with severe injuries (ISS > 15), no significant association was observed for the female sex on any outcome, including poor GOS ( aOR 0.89, 99% CI 0.64 to 1.24, *p* = 0.89) and poor mRS( aOR 0.87, 99% CI 0.62 to 1.21, *p* = 0.268) at discharge.

**Table 4:** Logistic regression model in predicting outcomes in all age groups

	aOR	*P* value	99% CI
**In-Hospital Mortality**			
**Age < 50**	3.26	< 0.001	2.54, 4.18
**Non -penetrating injuries**	139.55	< 0.001	110.73, 175.86
**Anatomical Location Head**	0.53	< 0.001	0.41, 0.68
**RR > 20**	0.4	< 0.001	0.31, 0.52
**GCS 3–8**	0.02	< 0.001	0.02, 0.03
**ISS 9–15**	0.71	0.001	0.55, 0.92
**Female**	1.24	0.022	0.98, 1.57
**Poor GOS at Discharge** ^**a**^			
**Age < 50**	0.40	< 0.001	0.36, 0.44
**Penetrating injuries**	0.33	< 0.001	0.23, 0.46
**Female**	0.97	0.003	0.82, 0.99
**ISS 9–15**	3.68	< 0.001	3.37, 4.03
**RR > 20**	2.87	< 0.001	2.55, 3.24
**GCS 9–12**	3.81	< 0.001	3.13, 4.64
**Poor mRS at Discharge** ^**b**^			
**Age < 50**	0.54	< 0.001	0.49, 0.59
**Penetrating injuries**	0.55	< 0.001	0.42, 0.71
**RR > 20**	1.59	< 0.001	1.40, 1.80
**Female**	0.98	0.593	0.90, 1.07
**GCS 13–15**	0.13	< 0.001	0.11, 0.16
**ISS 16–25**	3.97	< 0.001	3.50, 4.50
**ISS 9–15**	5.35	< 0.001	4.90, 5.84
**GCS 9–12**	0.37	< 0.001	0.28, 0.49

GOS, Glasgow Outcome Scale; MRS, Modified Rankin’s Scale; SBP, Systolic Blood Pressure; RR, Respiratory Rate; GCS, Glasgow Coma Scale; ISS, Injury Severity Score; aOR, adjusted Odds Ratio

^a^ Defined as GOS score of 2–3

^b^ Defined as mRS score of 4–5

**Table 5:** Logistic regression model in Predicting outcomes for age < 50 years

	aOR	*P* value	99% CI
**In-Hospital Mortality**			
**Female**	0.98	0.907	0.61, 1.56
**Anatomical Location Head**	0.48	< 0.001	0.29, 0.80
**RR > 20**	0.51	< 0.001	0.34, 0.78
**GCS 3–8**	0.01	< 0.001	0.01, 0.02
**ISS 9–15**	0.91	0.606	0.55, 1.49
**Poor GOS at Discharge** ^**a**^			
**Female**	0.63	< 0.001	0.51, 0.78
**ISS 9–15**	3.15	< 0.001	2.59, 3.82
**RR > 20**	3.97	< 0.001	3.25, 4.86
**GCS 9–12**	3.45	< 0.001	2.43, 4.87
**Poor mRS at Discharge** ^**b**^			
**Female**	0.72	< 0.001	0.62, 0.83
**RR > 20**	1.92	< 0.001	1.64, 2.26
**GCS 13–15**	0.06	< 0.001	0.05, 0.07
**ISS 16–25**	4.17	< 0.001	3.50, 4.96
**ISS 9–15**	4.83	< 0.001	4.21, 5.55
**GCS 9–12**	0.19	< 0.001	0.13, 0.26

GOS, Glasgow Outcome Scale; MRS, Modified Rankin’s Scale; SBP, Systolic Blood Pressure; RR, Respiratory Rate; GCS, Glasgow Coma Scale; ISS, Injury Severity Score; aOR, adjusted Odds Ratio

^a^ Defined as GOS score of 2–3

^b^ Defined as mRS score of 4–5

**Table 6:** Logistic regression model in Predicting outcomes for age < 50 years and ISS > 16

**Poor GOS at Discharge**			
	**aOR**	***P*** **value**	**99% CI**
**Female**	0.89	0.377	0.64, 1.24
**Mechanism** **penetrating**	0.19	0.002	0.05,0.75
**RR > 20**	2.34	< 0.001	1.76, 3.13
**GCS 9–12**	1.52	0.027	0.93, 2.48
**Poor mRS at Discharge**			
**Female**	0.87	0.268	0.62,1.21
**RR > 20**	1.48	< 0.001	1.10, 1.98
**GCS 13–15**	0.31	< 0.001	0.22, 0.43
**GCS 9–12**	0.72	0.099	0.42, 1.21

GOS, Glasgow Outcome Scale; MRS, Modified Rankin’s Scale; RR, Respiratory Rate; GCS, Glasgow Coma Scale; aOR, adjusted Odds Ratio

^a^ Defined as GOS score of 2–3

^b^ Defined as mRS score of 4–5

## Discussion

The difference in trauma outcomes between sexes has remained inconclusive despite extensive research. Our study contributes to this debate by examining a large sample from 12 Asian Pacific countries. In contrast to some regional studies that suggested better outcomes for Asian females, our results aligned more closely with studies from developed nations, showing no significant advantage for females in trauma mortalities [3, 16, 27]. Notably, studies in Taiwan and Korea with large sample sizes did not find improved female survival rates, thus supporting our findings [28, 29]. The inconsistencies in earlier research could be attributed to varying sample sizes and probably regional healthcare disparities.

Our study’s large and diverse sample size strengthens the validity of our findings. However, it is crucial to consider the complex interplay between the different healthcare systems and biological and social factors that influence trauma outcomes. Despite this complexity, our findings suggest a general pattern similar to that observed in developed and middle-income countries [30].

Specifically, the crude in-hospital mortality was higher in males ( 1.6% for males vs. 1.06% for females). When examining patients with ISS > 15, the mortality rate was 8.78% for females and 9.81% for males, which is lower than the Netherlands (18.5% for females and 17.5% for males ) [27]. The difference might be related to the higher severity of injury in the Netherlands, where about 50%of cases had an ISS > 15, compared to around 27% in our study.

Although the initial assessment showed a significant difference in mortality outcomes between the sexes, the regression model did not show any significant association between the female sex and reduced mortality. A similar pattern was observed in the subgroup analysis of < 50 years and with those with ISS > 15. This study challenges the notion that females have a survival advantage in severe injury cases [3].

To address the relatively low mortality rate compared to the overall size of the data, we explored the functional outcome as an additional measure. By analysing poor outcomes of GOS and MRS after excluding mortality, both univariate and multivariate analysis of all age groups revealed no significant difference in outcomes between sexes. However, stratification by age under 50 indicated that females are 37%(GOS) and 28%(mRS) less likely to be associated with poor functional outcomes than males. This association, however, was not observed when further stratified by ISS > 15, indicating that the protective effect of females diminishes with increasing injury severity. This finding is intriguing as, to the best of our knowledge, no previous comparison of GOS and mRS based on sex for trauma outcomes has been made.

Our findings align partially with other studies, which found a lower incidence of multi-organ failures in females aged 16–44 with an ISS > 16 but no difference in overall mortality [31, 32]. Additionally, a meta-analysis of 19 studies of trauma outcomes suggested that the protective benefit of sex decreases in those over 50 years [33]. Although we observed no significant difference in outcomes overall, the results for younger females with relatively less severe injuries (ISS < 16) raise the possibility that reproductive hormones could confer a protective advantage. This benefit appears attenuated with severe injuries or in post-menopausal females, highlighting the possible complexity of hormonal and immune responses in trauma. Further research is needed to clarify these mechanisms and their clinical implications.

The apparent significant mortality differences between sexes in the univariate analysis could be influenced by the injury pattern and mechanism. The major contributor of injuries in females was falls ( 44% vs. 31% in males), leading to the majority of lower extremities injuries, and hence the dominance of those with admission ISS < 16. This results in fewer mortalities among females ( one mortality to every 94 cases) compared to males (one mortality to every 62 cases), explaining the significant difference in mortality rates. Additionally, more female mortalities occur in the ISS < 16 groups ( 42% ) compared to males (31% ), with a significant difference between sexes in the ISS 9–15 group. The mortality in females could be attributed to age and comorbidities rather than sex itself. By controlling the age in the regression analysis, sex did not show a significant association with outcomes, except for functional outcomes in those under 50 years with ISS < 16.

In evaluating other characteristics, males generally sustained more critical injuries to vital anatomical regions—particularly the head, face, thorax, and abdomen—and presented with worse initial GCS and higher ISS (> 15) than females. Cultural factors, risk-taking behaviours, or greater mobility among males may influence this pattern. The elevated frequency of head, abdominal, and thoracic trauma in males also corresponds with higher trauma-related mortality, aligning with prior findings on injury severity in these regions [34]. Specifically, the study also reported hazard ratios for mortality of 4.5 for head injuries, 3.62 for abdominal injuries, and 1.36 for thoracic injuries. Further, abdominal and thoracic injuries were associated with a higher rate of massive blood transfusions, underscoring the gravity of such injuries and the need for timely interventions [35].

In the adjusted analysis of all age groups, excluding sex, other variables were strongly associated with in-hospital mortality, poor GOS, and poor mRS upon discharge. Among patients under 50 years, females showed a significantly inversely association with poor GOS and mRS upon discharge.

Interestingly, no significant difference in SBP < 90 was observed between sexes, contrasting with findings from previous large studies [32]. This discrepancy might stem from differences in study rigour regarding shock definition and the timing of BP measurement upon admission.

Another notable finding is the consistent association of poor functional outcomes with the high respiratory rate in unadjusted analysis. This is aligned with the other studies that identified initial RR and O2 saturation as good prognostic indicators in trauma [36].

In conclusion, this study advances previous research by comprehensively analysing trauma outcomes across a large, diverse sample in the Asia-Pacific region. Overall, mortality and functional outcomes do not significantly differ between sexes. The higher rate of poor outcomes observed in males is linked to a greater incidence of trauma in more severe anatomical locations, which generally leads to worse outcomes. Although the subgroup analysis of young females shows better functional outcomes at discharge, it is only limited to ISS < 16, and hence, further research is necessary to validate these results. These findings underscore the importance of considering sex and age in trauma care and research, potentially leading to more personalised approaches to treatment and rehabilitation.

### Limitation

Although we provided comprehensive demographic, physiological, and anatomical data—including injury location, mechanism, and outcomes by gender—certain details, such as more precise organ injury grades, comorbidities, and timing of interventions, were not fully explored. These factors may further illuminate the interplay between patient characteristics, injury patterns, and outcomes.

Additionally, the countries’ diversity introduces variability in healthcare systems, which must be considered when interpreting the outcomes. Incomplete or missing data, although minimised through computational methods, could introduce bias. Furthermore, the study’s focus on 12 Asian Pacific countries limits the generalizability of the findings to the entire region. Lastly, as this study is observational, causality cannot be established.

## Data Availability

No datasets were generated or analysed during the current study.

## Abbreviations

PATOS: The Pan-Asia trauma outcomes study
ISS: Injury severity score
GOS: Glasgow outcome scale
MRS: Modified Rankin’s Scale
GCS: Glasgow coma scale
SBP: Systolic blood pressure
RR: Respiratory rate
